# Supplementary material for: Deep phenotyping of 34,128 adult patients hospitalised with COVID-19 in an international network study
Source: Nat Commun. 2020 Oct 6;11:5009. doi: 10.1038/s41467-020-18849-z (PMC7538555; doi:10.1038/s41467-020-18849-z)
Supplement: Supplementary file 1 — Supplementary Information [file 41467_2020_18849_MOESM1_ESM.pdf]

Supplementary Information

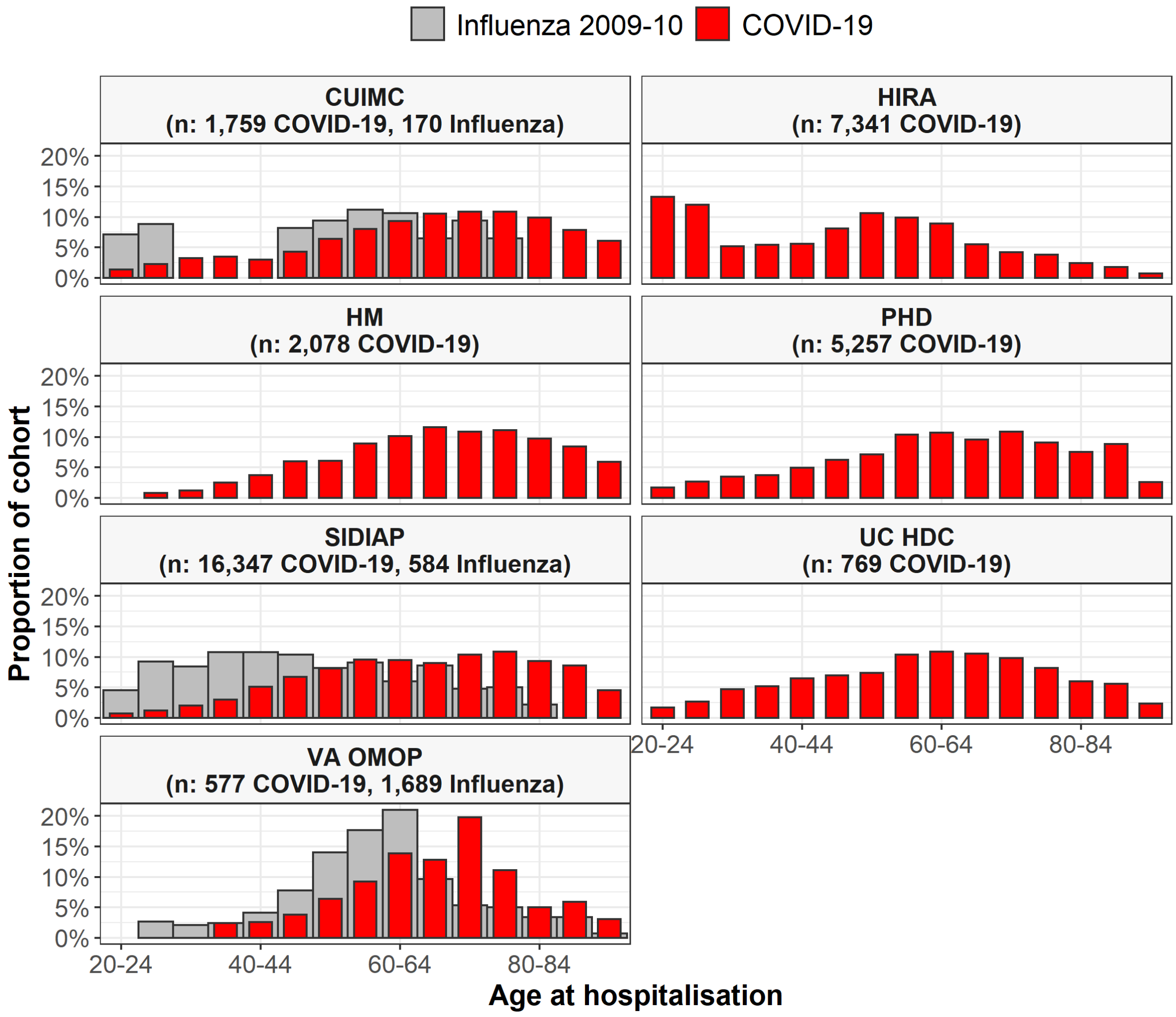

Supplementary Figure 1. Age of patients hospitalised with COVID-19 and of patients hospitalised with influenza

Individuals hospitalised with COVID-19 between December 2019 and April 2020 compared with those hospitalised with influenza between September 2009 to April 2010. Proportion of cohorts by 5-year age groups, with counts of less than 10 omitted. CUIMC: Columbia University Irving Medical Center; HIRA: Health Insurance Review & Assessment; HM: HM Hospitales; PHD: Premier Healthcare Database; SIDIAP: The Information System for Research in Primary Care; UC HDC: University of Colorado Health Data Compass; VA OMOP: Department of Veterans Affairs.

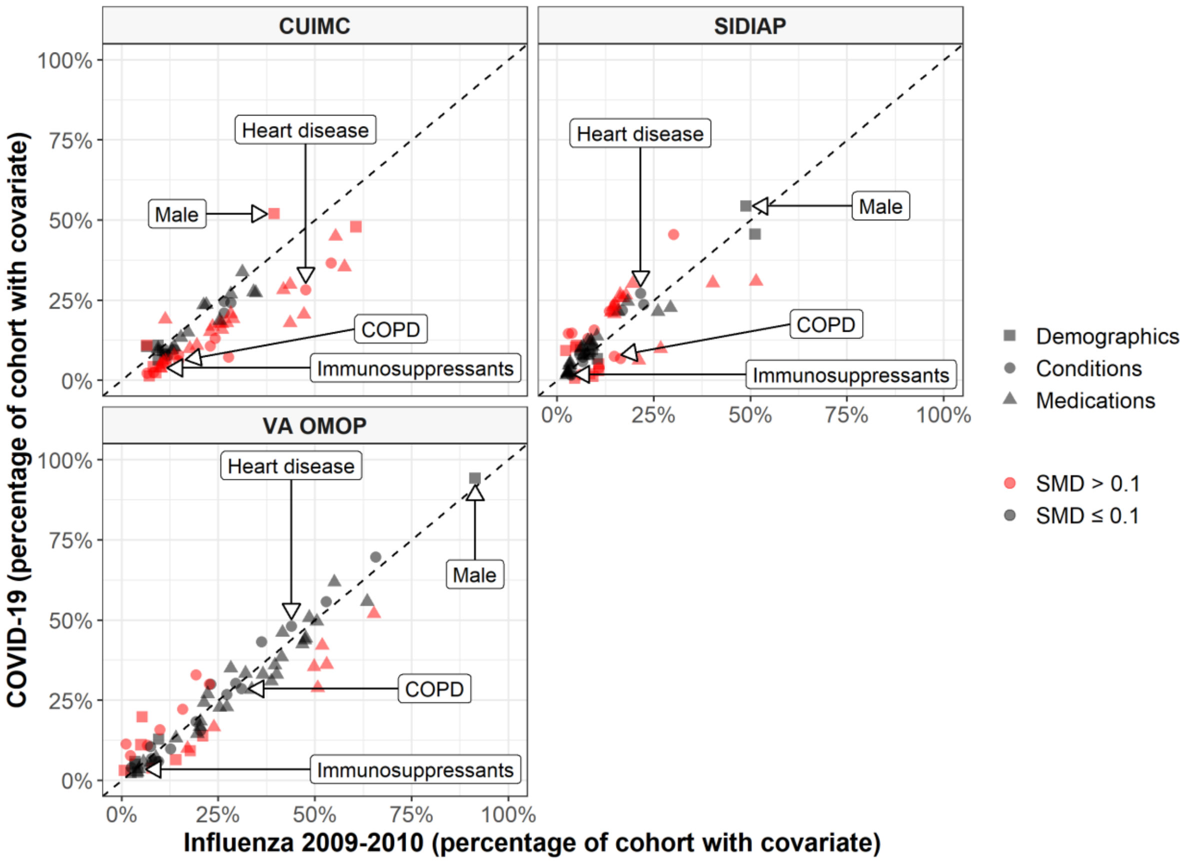

Supplementary Figure 2. Characteristics of COVID-19 patients compared to 2009-2010 Influenza patients

The plot compares demographics (age and sex), conditions (recorded over the year prior and up to day of hospitalisation), and medications (1) from a year prior up to, and including, day of hospitalisation, 2) from 30 days prior up to, and including, day of hospitalisation, and 3) on day of hospitalisation). Each dot represents one of these covariates with the colour indicating the absolute value of the standardised mean difference (SMD), with a SMD above 0.1 taken to indicate a difference in the prevalence of a particular covariate. The proportion male, with heart disease, with chronic obstructive pulmonary disease (COPD) and taking immunosuppressants (over the 30 days prior up to, and including, day of hospitalisation) are shown for illustration. CUIMC: Columbia University Irving Medical Center; SIDIAP: The Information System for Research in Primary Care; VA OMOP: Department of Veterans Affairs.

Supplementary Table 1. Characteristics of individuals hospitalised with COVID-19 compared with those hospitalised with influenza in 2015-2019

	CUIMC COVID-19	CUIMC Influenza 2014-19	CUIMC SMD	PHD COVID-19	PHD Influenza 2014-19	PHD SMD	SIDIAP COVID-19	SIDIAP Influenza 2014-19	SIDIAP SMD	UC HDC COVID-19	UC HDC Influenza 2014-19	UC HDC SMD	VA OMOP COVID-19	VA OMOP Influenza 2014-19	VA OMOP SMD
sex: male	52.0%	43.1%	0.09	52.4%	44.1%	0.08	54.4%	52.9%	0.01	53.7%	44.9%	0.09	94.3%	94.1%	0.00
sex: female	47.9%	56.9%	-0.09	47.6%	55.9%	-0.08	45.6%	47.1%	-0.02	46.3%	54.9%	-0.09	5.7%	5.9%	-0.01
age group: 15-19	<0.6%	1.2%		0.4%	0.4%	0.00	0.1%	0.6%	-0.06		0.4%				
age group: 20-24	1.4%	3.2%	-0.08	1.7%	1.5%	0.01	0.7%	1.3%	-0.04	1.7%	2.6%	-0.04	<1.7%		
age group: 25-29	2.3%	4.0%	-0.07	2.7%	2.2%	0.02	1.2%	2.0%	-0.04	2.7%	3.4%	-0.03	<1.7%	0.4%	
age group: 30-34	3.2%	3.8%	-0.02	3.5%	2.4%	0.05	2.0%	2.7%	-0.03	4.7%	3.6%	0.04	<1.7%	0.8%	
age group: 35-39	3.5%	3.1%	0.02	3.7%	2.6%	0.04	3.0%	3.9%	-0.03	5.2%	3.9%	0.04	2.4%	1.0%	0.08
age group: 40-44	3.0%	3.0%	0.00	4.9%	2.9%	0.07	5.1%	4.9%	0.01	6.5%	3.9%	0.08	2.6%	1.1%	0.08
age group: 45-49	4.3%	3.8%	0.02	6.2%	4.3%	0.06	6.7%	5.3%	0.04	7.0%	4.9%	0.06	3.8%	2.1%	0.07
age group: 50-54	6.4%	5.8%	0.02	7.1%	5.9%	0.03	8.1%	6.0%	0.06	7.4%	6.2%	0.03	6.4%	3.7%	0.09
age group: 55-59	8.0%	6.6%	0.04	10.4%	9.3%	0.02	9.6%	7.0%	0.06	10.4%	8.5%	0.04	9.2%	7.5%	0.04

age group: 60-64	9.3%	8.6%	0.02	10.7%	11.2%	-0.01	9.5%	7.4%	0.05	10.9%	10.6%	0.01	13.9%	12.4%	0.03
age group: 65-69	10.5%	9.8%	0.02	9.6%	10.9%	-0.03	9.0%	9.5%	-0.01	10.5%	11.0%	-0.01	12.8%	19.1%	-0.11
age group: 70-74	10.9%	9.3%	0.04	10.9%	11.9%	-0.02	10.4%	10.9%	-0.01	9.8%	10.0%	0.00	19.8%	19.7%	0.00
age group: 75-79	10.9%	10.3%	0.01	9.1%	11.2%	-0.05	10.9%	10.7%	0.00	8.2%	8.9%	-0.02	11.1%	9.6%	0.03
age group: 80-84	9.9%	9.7%	0.00	7.5%	9.5%	-0.05	9.3%	12.6%	-0.07	6.0%	8.6%	-0.07	5.0%	8.2%	-0.09
age group: 85-89	7.9%	8.1%	0.00	8.8%	13.8%	-0.11	8.6%	8.7%	0.00	5.6%	6.6%	-0.03	5.9%	7.3%	-0.04
age group: 90-94	6.1%	6.5%	-0.01	2.6%	0.1%	0.15	4.5%	5.2%	-0.02	2.3%	5.1%	-0.10	3.1%	5.3%	-0.08
age group: 95-99	1.8%	2.4%	-0.03				1.2%	1.2%	0.00	<1.3%	1.3%		<1.7%	1.6%	
age group: 00-04	<0.6%	0.6%					0.1%	<0.3%		<1.3%	<0.3%			0.1%	
age group: 05-10															
Charlson comorbidity index	4.0	5.7	-0.29	3.1	3.1	0.00	1.5	3.5	-0.59	3.5	4.3	-0.16	5.4	5.9	-0.09
Anemia	13.1%	25.5%	-0.20	30.2%	26.8%	0.05	14.6%			29.9%	37.6%	-0.09	30.0%	27.3%	0.04
Anxiety disorder	5.5%	9.7%	-0.11	18.0%	18.6%	-0.01	23.7%	20.2%	0.05	18.2%	26.9%	-0.13	30.3%	29.0%	0.02
Asthma	7.2%	21.2%	-0.26	14.6%	14.0%	0.01	6.9%	12.2%	-0.12	13.8%	24.3%	-0.17	9.9%	8.3%	0.04
Atrial fibrillation	8.4%	20.0%	-0.22	18.1%	22.6%	-0.07	8.2%	16.4%	-0.17	12.0%	18.1%	-0.11	15.8%	20.8%	-0.08
Chronic liver disease	2.6%	5.2%	-0.09	3.0%	2.8%	0.01	1.9%	7.0%	-0.17	2.3%	5.3%	-0.11	6.8%	7.5%	-0.02
COPD	6.1%	18.3%	-0.25	24.5%	36.6%	-0.15	7.6%	23.2%	-0.28	12.6%	31.1%	-0.28	28.6%	43.0%	-0.17

Dementia	8.5%	9.4%	-0.02	9.2%	9.9%	-0.02	5.6%	5.3%	0.01	7.4%	8.1%	-0.02	7.8%	10.2%	-0.06
Diabetes mellitus	24.2%	32.2%	-0.11	35.8%	34.0%	0.02	21.6%	29.3%	-0.11	35.2%	31.8%	0.04	43.3%	43.1%	0.00
Gastroesophageal reflux disease	9.6%	16.4%	-0.13	24.8%	25.2%	-0.01	9.5%	6.2%	0.08	23.4%	35.0%	-0.15	26.9%	29.1%	-0.03
Heart disease	28.3%	53.0%	-0.27	48.6%	54.2%	-0.06	27.1%	49.1%	-0.25	39.0%	55.8%	-0.17	48.2%	58.2%	-0.10
Heart failure	10.7%	25.8%	-0.25	24.8%	28.1%	-0.05	5.9%	20.0%	-0.28	12.9%	23.1%	-0.17	22.2%	25.7%	-0.05
Hyperlipidemia	24.7%	33.1%	-0.11	46.0%	45.1%	0.01	23.7%	39.8%	-0.20	38.0%	43.0%	-0.06	55.8%	58.5%	-0.03
Hypertensive disorder	36.6%	50.6%	-0.15	47.1%	41.1%	0.06	45.6%	55.8%	-0.10	57.3%	62.5%	-0.05	69.7%	71.8%	-0.02
Insomnia	3.1%	5.6%	-0.08	4.4%	3.3%	0.04	14.6%	12.6%	0.04	7.4%	10.4%	-0.07	10.9%	9.9%	0.02
Ischemic heart disease	8.0%	15.2%	-0.15	16.0%	16.8%	-0.01	7.3%	17.1%	-0.20	13.5%	17.5%	-0.07	15.6%	21.3%	-0.09
Low back pain	6.8%	8.9%	-0.05	5.0%	2.7%	0.08	21.9%	25.7%	-0.06	11.1%	14.0%	-0.06	30.0%	26.4%	0.05
Malignant neoplastic disease	7.7%	17.5%	-0.20	9.4%	8.5%	0.02	15.8%	23.5%	-0.12	10.8%	19.3%	-0.15	18.4%	21.4%	-0.05
Osteoarthritis of knee	4.6%	4.4%	0.01	2.0%	1.3%	0.04	14.8%	11.9%	0.06	5.1%	4.7%	0.01	11.4%	6.6%	0.11
Osteoarthritis of hip	3.0%	3.2%	-0.01	1.1%	0.5%	0.05	5.1%	3.8%	0.04	2.6%	2.1%	0.02	2.8%	1.8%	0.05
Peripheral vascular disease	4.7%	5.9%	-0.04	7.5%	6.2%	0.04	3.9%	24.8%	-0.39	7.4%	6.9%	0.01	10.6%	12.7%	-0.04
Renal impairment	21.1%	34.9%	-0.18	37.5%	36.9%	0.01	13.1%	26.9%	-0.22	37.2%	39.5%	-0.03	32.9%	32.2%	0.01

Venous thrombosis	2.3%	7.7%	-0.17	2.0%	1.8%	0.01	1.5%	2.6%	-0.05	6.6%	7.7%	-0.03	4.0%	4.0%	0.00
Viral hepatitis	2.3%	3.9%	-0.06	2.5%	2.4%	0.00	1.7%	4.8%	-0.12	2.6%	3.9%	-0.05	5.9%	6.3%	-0.01
Antineoplastic and immunomodulating agents (year up to, and including, index date)	9.6%	21.3%	-0.21	3.8%	2.6%	0.05	3.4%	8.9%	-0.16	12.9%	23.0%	-0.17	13.2%	13.5%	-0.01
Antineoplastic and immunomodulating agents (30 days up to, and including, index date)	6.1%	17.0%	-0.23	2.6%	2.4%	0.01	2.7%	7.7%	-0.15	9.6%	18.3%	-0.16	7.1%	8.5%	-0.04
Antineoplastic and immunomodulating agents (on index date)	4.9%	15.0%	-0.23	2.1%	2.0%	0.00	2.5%	7.6%	-0.16	8.2%	16.7%	-0.17	4.9%	6.7%	-0.05
Agents acting on the renin-angiotensin system (year up to, and including, index date)	27.3%	36.3%	-0.11	15.3%	12.7%	0.05	30.2%	38.2%	-0.10	39.8%	40.9%	-0.01	49.6%	50.8%	-0.01
Agents acting on the renin-angiotensin system (30 days up to, and including, index date)	18.0%	25.1%	-0.11	8.8%	11.8%	-0.07	26.6%	33.7%	-0.09	33.0%	35.8%	-0.03	38.5%	41.0%	-0.03
Agents acting on the renin-angiotensin system (on index date)	15.9%	23.8%	-0.13	7.4%	11.0%	-0.08	25.7%	32.5%	-0.09	31.5%	34.9%	-0.04	33.1%	38.8%	-0.07

Antiepileptics (year up to, and including, index date)	19.0%	24.9%	-0.09	16.7%	15.9%	0.01	10.6%	15.3%	-0.09	27.2%	33.0%	-0.07	33.4%	37.4%	-0.05
Antiepileptics (30 days up to, and including, index date)	9.9%	17.0%	-0.14	12.2%	15.3%	-0.06	8.1%	12.1%	-0.09	21.6%	27.1%	-0.08	22.7%	30.0%	-0.10
Antiepileptics (on index date)	9.1%	15.7%	-0.13	11.3%	14.8%	-0.07	7.7%	11.7%	-0.09	20.2%	26.5%	-0.09	16.6%	28.4%	-0.18
Antiinflammatory and antirheumatic products (year up to index date)	23.6%	34.2%	-0.14	17.5%	14.9%	0.05	30.9%	34.8%	-0.05	52.9%	74.8%	-0.19	46.1%	36.0%	0.11
Antiinflammatory and antirheumatic products (30 days up to, and including, index date)	10.5%	20.0%	-0.17	10.2%	14.2%	-0.08	9.9%	15.1%	-0.10	38.6%	62.3%	-0.24	16.8%	17.3%	-0.01
Antiinflammatory and antirheumatic products (on index date)	7.8%	17.1%	-0.19	7.9%	12.3%	-0.10	6.3%	11.1%	-0.12	35.1%	59.3%	-0.25	9.9%	13.2%	-0.07
Antithrombotic agents (year up to, and including, index date)	44.9%	75.0%	-0.27	50.1%	49.0%	0.01	25.7%	39.1%	-0.17	72.0%	75.9%	-0.03	55.8%	72.0%	-0.14
Antithrombotic agents (30 days up to, and including, index date)	29.9%	64.8%	-0.36	40.4%	47.7%	-0.08	21.9%	35.0%	-0.17	67.5%	68.4%	-0.01	42.1%	61.4%	-0.19

Antithrombotic agents (on index date)	28.3%	63.5%	-0.37	38.4%	46.7%	-0.09	21.3%	34.3%	-0.17	66.8%	67.3%	0.00	35.5%	59.0%	-0.24
Beta blocking agents (year up to, and including, index date)	26.9%	39.5%	-0.15	26.7%	25.7%	0.01	13.8%	22.9%	-0.15	32.8%	42.7%	-0.11	44.4%	53.3%	-0.09
Beta blocking agents (30 days up to, and including, index date)	16.8%	31.0%	-0.21	19.3%	24.6%	-0.08	12.4%	20.3%	-0.14	24.7%	35.6%	-0.14	36.0%	46.0%	-0.11
Beta blocking agents (on index date)	15.2%	29.9%	-0.22	17.8%	23.8%	-0.09	11.9%	19.7%	-0.14	23.8%	34.4%	-0.14	31.0%	44.5%	-0.16
Calcium channel blockers (year up to, and including, index date)	23.5%	32.5%	-0.12	17.4%	15.0%	0.04	12.4%	18.9%	-0.12	24.3%	27.8%	-0.05	35.0%	34.4%	0.01
Calcium channel blockers (30 days up to, and including, index date)	15.0%	22.5%	-0.12	11.1%	14.2%	-0.06	10.5%	15.5%	-0.10	20.5%	22.2%	-0.03	27.0%	26.8%	0.00
Calcium channel blockers (on index date)	13.4%	21.1%	-0.13	9.7%	13.4%	-0.08	10.1%	15.0%	-0.10	19.9%	21.3%	-0.02	24.3%	25.5%	-0.02
Diuretics (year up to, and including, index date)	27.6%	42.4%	-0.18	22.7%	19.4%	0.05	24.6%	38.9%	-0.18	33.3%	42.8%	-0.11	42.6%	47.1%	-0.05
Diuretics (30 days up to, and including, index date)	19.2%	32.2%	-0.18	15.6%	18.2%	-0.04	21.5%	34.4%	-0.17	27.4%	35.6%	-0.10	33.1%	37.1%	-0.05

Diuretics (on index date)	17.7%	30.2%	-0.18	14.1%	17.2%	-0.06	20.7%	33.6%	-0.17	26.4%	34.6%	-0.10	28.4%	34.7%	-0.08
Drugs for acid related disorders (year up to, and including, index date)	35.4%	60.0%	-0.25	34.5%	31.4%	0.04	30.4%	60.3%	-0.31	53.6%	67.7%	-0.13	52.0%	60.7%	-0.08
Drugs for acid related disorders (30 days up to, and including, index date)	20.6%	45.2%	-0.30	23.7%	30.0%	-0.09	22.7%	51.6%	-0.34	44.5%	58.7%	-0.14	36.2%	47.5%	-0.12
Drugs for acid related disorders (on index date)	17.9%	42.5%	-0.32	21.8%	28.5%	-0.09	21.5%	50.5%	-0.34	42.4%	56.8%	-0.14	28.9%	44.8%	-0.19
Immunosuppressants (year up to, and including, index date)	5.6%	13.4%	-0.18	2.2%	1.4%	0.04	2.3%	7.0%	-0.15	7.5%	11.5%	-0.09	5.7%	5.4%	0.01
Immunosuppressants (30 days up to, and including, index date)	3.9%	11.5%	-0.19	1.6%	1.3%	0.02	1.9%	6.3%	-0.15	6.1%	9.6%	-0.09	3.5%	4.1%	-0.02
Immunosuppressants (on index date)	3.7%	10.7%	-0.18	1.5%	1.3%	0.01	1.7%	6.2%	-0.16	5.9%	9.3%	-0.09	2.6%	3.7%	-0.04
Insulins and analogues (year up to, and including, index date)	17.8%	31.7%	-0.20	19.2%	18.0%	0.02	5.5%	11.0%	-0.14	31.9%	28.8%	0.04	22.9%	30.0%	-0.10
Insulins and analogues (30 days up to, and including, index date)	10.9%	22.3%	-0.20	13.9%	17.3%	-0.06	4.9%	10.5%	-0.14	26.9%	24.7%	0.03	18.4%	24.0%	-0.09

Insulins and analogues (on index date)	9.9%	21.2%	-0.20	13.1%	16.7%	-0.07	4.7%	10.3%	-0.14	25.4%	23.9%	0.02	14.6%	22.6%	-0.13
Lipid modifying agents (year up to, and including, index date)	33.8%	43.6%	-0.11	27.6%	27.2%	0.01	26.8%	36.3%	-0.12	41.7%	43.6%	-0.02	61.9%	63.5%	-0.01
Lipid modifying agents (30 days up to, and including, index date)	20.8%	35.2%	-0.19	21.0%	26.4%	-0.08	23.6%	32.8%	-0.12	36.4%	39.1%	-0.03	50.8%	54.5%	-0.04
Lipid modifying agents (on index date)	18.7%	34.0%	-0.21	19.9%	25.8%	-0.09	23.0%	32.4%	-0.13	35.6%	38.2%	-0.03	43.8%	52.7%	-0.09

CUIMC: Columbia University Irving Medical Center; HIRA: Health Insurance Review & Assessment; HM: HM Hospitales; PHD: Premier Healthcare Database; SIDIAP: The Information System for Research in Primary Care; UC HDC: University of Colorado Health Data Compass; VA OMOP: Department of Veterans Affairs. COPD: Chronic obstructive pulmonary disease; GERD: Gastroesophageal reflux disease. Exact proportions have not been reported where counts were less than 10.

Supplementary Table 2. Characteristics of individuals hospitalised with COVID-19 compared with those hospitalised with influenza in 2009-2010

	CUIMC COVID-19	CUIMC Influenza 2009-10	CUIMC SMD	SIDIAP COVID-19	SIDIAP Influenza 2009-10	SIDIAP SMD	VA OMOP COVID-19	VA OMOP Influenza 2009-10	VA OMOP SMD
sex: male	52.0%	39.4%	0.13	54.4%	48.8%	0.06	94.3%	91.4%	0.02
sex: female	47.9%	60.6%	-0.12	45.6%	51.2%	-0.06	5.7%	8.6%	-0.08
age group: 15-19	<0.6%	<5.9%		0.1%	<1.7%				
age group: 20-24	1.4%	7.1%	-0.20	0.7%	4.5%	-0.17	<1.7%	<0.6%	
age group: 25-29	2.3%	8.8%	-0.19	1.2%	9.2%	-0.25	<1.7%	2.7%	
age group: 30-34	3.2%	<5.9%		2.0%	8.4%	-0.20	<1.7%	2.1%	
age group: 35-39	3.5%	<5.9%		3.0%	10.8%	-0.21	2.4%	2.4%	0.00
age group: 40-44	3.0%	<5.9%		5.1%	10.8%	-0.14	2.6%	4.1%	-0.06
age group: 45-49	4.3%	8.2%	-0.11	6.7%	10.4%	-0.09	3.8%	7.8%	-0.12
age group: 50-54	6.4%	9.4%	-0.08	8.1%	8.2%	0.00	6.4%	14.0%	-0.17
age group: 55-59	8.0%	11.2%	-0.07	9.6%	9.1%	0.01	9.2%	17.7%	-0.16
age group: 60-64	9.3%	10.6%	-0.03	9.5%	6.0%	0.09	13.9%	21.0%	-0.12
age group: 65-69	10.5%	6.5%	0.10	9.0%	8.6%	0.01	12.8%	9.6%	0.07
age group: 70-74	10.9%	9.4%	0.03	10.4%	4.8%	0.14	19.8%	5.3%	0.29
age group: 75-79	10.9%	6.5%	0.11	10.9%	5.0%	0.15	11.1%	5.0%	0.15
age group: 80-84	9.9%	<5.9%		9.3%	2.2%	0.21	5.0%	3.4%	0.06
age group: 85-89	7.9%	<5.9%		8.6%	<1.7%		5.9%	3.4%	0.08
age group: 90-94	6.1%	<5.9%		4.5%	<1.7%		3.1%	0.7%	0.12
age group: 95-99	1.8%	<5.9%		1.2%			<1.7%	<0.6%	
age group: 00-04	<0.6%	<5.9%		0.1%					
age group: 05-10									
charlson index	4.0	4.8	-0.13	1.5	1.6	-0.03	5.4	4.2	0.24

Anemia	13.1%	24.1%	-0.18	14.6%			30.0%	23.0%	0.10
Anxiety disorder	5.5%	10.6%	-0.13	23.7%	14.9%	0.14	30.3%	29.4%	0.01
Asthma	7.2%	27.6%	-0.35	6.9%	16.4%	-0.20	9.9%	12.6%	-0.06
Atrial fibrillation	8.4%	11.2%	-0.06	8.2%	5.8%	0.06	15.8%	9.9%	0.12
Chronic liver disease	2.6%	8.2%	-0.17	1.9%	6.0%	-0.15	6.8%	7.6%	-0.02
COPD	6.1%	14.7%	-0.19	7.6%	14.7%	-0.15	28.6%	31.0%	-0.03
Dementia	8.5%	<5.9%		5.6%	<1.7%		7.8%	2.2%	0.18
Diabetes mellitus	24.2%	28.2%	-0.06	21.6%	13.5%	0.14	43.3%	36.1%	0.08
Gastroesophageal reflux disease	9.6%	13.5%	-0.08	9.5%	<1.7%		26.9%	27.2%	0.00
Heart disease	28.3%	47.6%	-0.22	27.1%	21.6%	0.08	48.2%	43.9%	0.04
Heart failure	10.7%	22.9%	-0.21	5.9%	6.5%	-0.02	22.2%	15.7%	0.11
Hyperlipidemia	24.7%	26.5%	-0.03	23.7%	22.4%	0.02	55.8%	52.9%	0.03
Hypertensive disorder	36.6%	54.1%	-0.18	45.6%	30.1%	0.18	69.7%	65.7%	0.03
Insomnia	3.1%	<5.9%		14.6%	2.9%	0.28	10.9%	6.6%	0.10
Ischemic heart disease	8.0%	14.7%	-0.14	7.3%	6.7%	0.02	15.6%	20.3%	-0.08
Low back pain	6.8%	11.8%	-0.12	21.9%	16.8%	0.08	30.0%	22.6%	0.10
Malignant neoplastic disease	7.7%	11.8%	-0.09	15.8%	9.6%	0.12	18.4%	19.2%	-0.01
Osteoarthritis of knee	4.6%	<5.9%		14.8%	3.8%	0.26	11.4%	1.1%	0.29
Osteoarthritis of hip	3.0%			5.1%	<1.7%		2.8%		
Peripheral vascular disease	4.7%	<5.9%		3.9%	10.8%	-0.18	10.6%	7.4%	0.08
Renal impairment	21.1%	26.5%	-0.08	13.1%	7.9%	0.11	32.9%	19.2%	0.19
Venous thrombosis	2.3%	<5.9%		1.5%	<1.7%		4.0%	2.5%	0.06
Viral hepatitis	2.3%	6.5%	-0.14	1.7%	3.6%	-0.08	5.9%	9.5%	-0.09
Antineoplastic and immunomodulating agents (year up to index date)	9.6%	12.9%	-0.07	3.4%	3.6%	-0.01	13.2%	14.0%	-0.02
Antineoplastic and immunomodulating agents (30 days up to index date)	6.1%	11.2%	-0.12	2.7%	2.7%	0.00	7.1%	8.8%	-0.04

Antineoplastic and immunomodulating agents (on index date)	4.9%	10.0%	-0.13	2.5%	2.6%	0.00	4.9%	7.2%	-0.07
Agents acting on the renin-angiotensin system (year up to index date)	27.3%	34.7%	-0.09	30.2%	19.5%	0.15	49.6%	50.4%	-0.01
Agents acting on the renin-angiotensin system (30 days up to index date)	18.0%	27.1%	-0.14	26.6%	17.8%	0.13	38.5%	41.3%	-0.03
Agents acting on the renin-angiotensin system (on index date)	15.9%	25.9%	-0.15	25.7%	17.0%	0.13	33.1%	40.1%	-0.08
Antiepileptics (year up to index date)	19.0%	11.2%	0.14	10.6%	9.1%	0.03	33.4%	32.0%	0.02
Antiepileptics (30 days up to index date)	9.9%	9.4%	0.01	8.1%	7.7%	0.01	22.7%	25.3%	-0.04
Antiepileptics (on index date)	9.1%	9.4%	-0.01	7.7%	6.7%	0.03	16.6%	23.8%	-0.11
Antiinflammatory and antirheumatic products (year up to index date)	23.6%	21.8%	0.03	30.9%	51.4%	-0.23	46.1%	41.6%	0.05
Antiinflammatory and antirheumatic products (30 days up to index date)	10.5%	13.5%	-0.06	9.9%	26.7%	-0.28	16.8%	20.2%	-0.06
Antiinflammatory and antirheumatic products (on index date)	7.8%	13.5%	-0.12	6.3%	21.1%	-0.28	9.9%	17.0%	-0.14
Antithrombotic agents (year up to index date)	44.9%	55.3%	-0.10	25.7%	16.3%	0.14	55.8%	63.5%	-0.07
Antithrombotic agents (30 days up to index date)	29.9%	43.5%	-0.16	21.9%	14.7%	0.12	42.1%	51.8%	-0.10
Antithrombotic agents (on index date)	28.3%	41.8%	-0.16	21.3%	13.9%	0.12	35.5%	49.7%	-0.15
Beta blocking agents (year up to index date)	26.9%	28.2%	-0.02	13.8%	10.3%	0.07	44.4%	47.6%	-0.03
Beta blocking agents (30 days up to index date)	16.8%	23.5%	-0.11	12.4%	8.7%	0.08	36.0%	39.6%	-0.04
Beta blocking agents (on index date)	15.2%	22.9%	-0.12	11.9%	8.7%	0.07	31.0%	38.7%	-0.09
Calcium channel blockers (year up to index date)	23.5%	21.2%	0.03	12.4%	7.9%	0.10	35.0%	28.2%	0.09
Calcium channel blockers (30 days up to index date)	15.0%	17.1%	-0.04	10.5%	6.7%	0.09	27.0%	22.2%	0.07

Calcium channel blockers (on index date)	13.4%	15.3%	-0.04	10.1%	6.2%	0.10	24.3%	21.3%	0.04
Diuretics (year up to index date)	27.6%	34.1%	-0.08	24.6%	18.2%	0.10	42.6%	46.7%	-0.04
Diuretics (30 days up to index date)	19.2%	28.8%	-0.14	21.5%	15.8%	0.09	33.1%	36.5%	-0.04
Diuretics (on index date)	17.7%	25.9%	-0.12	20.7%	14.7%	0.10	28.4%	33.6%	-0.07
Drugs for acid related disorders (year up to index date)	35.4%	57.6%	-0.23	30.4%	40.2%	-0.12	52.0%	65.2%	-0.12
Drugs for acid related disorders (30 days up to index date)	20.6%	47.1%	-0.32	22.7%	29.3%	-0.09	36.2%	53.0%	-0.18
Drugs for acid related disorders (on index date)	17.9%	43.5%	-0.33	21.5%	26.0%	-0.07	28.9%	50.7%	-0.24
Immunosuppressants (year up to index date)	5.6%	10.6%	-0.12	2.3%	2.9%	-0.03	5.7%	5.6%	0.00
Immunosuppressants (30 days up to index date)	3.9%	10.0%	-0.16	1.9%	2.6%	-0.03	3.5%	4.4%	-0.03
Immunosuppressants (on index date)	3.7%	10.0%	-0.17	1.7%	2.4%	-0.03	2.6%	4.0%	-0.05
Insulins and analogues (year up to index date)	17.8%	25.3%	-0.11	5.5%	3.3%	0.07	22.9%	27.1%	-0.06
Insulins and analogues (30 days up to index date)	10.9%	19.4%	-0.15	4.9%	3.3%	0.06	18.4%	20.3%	-0.03
Insulins and analogues (on index date)	9.9%	17.6%	-0.15	4.7%	3.1%	0.06	14.6%	19.5%	-0.08
Lipid modifying agents (year up to index date)	33.8%	31.2%	0.03	26.8%	16.3%	0.16	61.9%	55.0%	0.06
Lipid modifying agents (30 days up to index date)	20.8%	28.2%	-0.11	23.6%	14.9%	0.14	50.8%	48.5%	0.02
Lipid modifying agents (on index date)	18.7%	25.3%	-0.10	23.0%	14.4%	0.14	43.8%	47.3%	-0.04

CUIMC: Columbia University Irving Medical Center; HIRA: Health Insurance Review & Assessment; HM: HM Hospitales; PHD: Premier Healthcare Database; SIDIAP: The Information System for Research in Primary Care; UC HDC: University of Colorado Health Data Compass; VA OMOP: Department of Veterans Affairs. COPD: Chronic obstructive pulmonary disease; GERD: Gastroesophageal reflux disease. Exact proportions have not been reported where counts were less than 10.

Supplementary Methods.

Identifying a COVID-19 hospitalisation

The below tables summarise the concepts used to identify a COVID-19 hospitalisation. Alongside a hospitalisation visit, one of these codes needed to be observed. The full description of the logic used is provided at https://atlas.ohdsi.org/#/cohortdefinition/108. (Similarly, the full description of the logic used to identify hospitalisations with influenza is provided at https://atlas.ohdsi.org/#/cohortdefinition/117).

COVID-19 condition codes

Id	Name	Vocabulary
37311061	Disease caused by 2019-nCoV	SNOMED
37310287	Myocarditis caused by 2019 novel coronavirus	SNOMED
37310286	Infection of upper respiratory tract caused by 2019 novel coronavirus	SNOMED
37310285	Pneumonia caused by 2019 novel coronavirus	SNOMED
37310284	Encephalopathy caused by 2019 novel coronavirus	SNOMED
37310283	Gastroenteritis caused by 2019 novel coronavirus	SNOMED
37310254	Otitis media caused by 2019 novel coronavirus	SNOMED
756081	Infection of lower respiratory tract due to COVID-19	OMOP Extension
756061	Asymptomatic COVID-19	OMOP Extension
756044	Acute respiratory distress syndrome (ARDS) due to COVID-19	OMOP Extension
756039	Respiratory infection due to COVID-19	OMOP Extension
756031	Bronchitis due to COVID-19	OMOP Extension
756023	Acute bronchitis due to COVID-19	OMOP Extension

COVID-19 specific testing - Positive

Id	Name	Vocabulary
37310282	2019 novel coronavirus detected	SNOMED

COVID-19 specific testing (note, these required a corresponding value as concept of: Detected, Positive, Positive, or Present)

Id	Name	Vocabulary
40218805	Testing for SARS-CoV-2 in CDC laboratory	HCPCS
40218804	Testing for SARS-CoV-2 in non-CDC laboratory	HCPCS
37310258	Measurement of 2019 novel coronavirus antibody	SNOMED
37310257	Measurement of 2019 novel coronavirus antigen	SNOMED
37310255	Detection of 2019 novel coronavirus using polymerase chain reaction technique	SNOMED
757686	SARS-CoV-2 (COVID19) IgA+IgM [Presence] in Serum or Plasma by Immunoassay	LOINC
757680	SARS-CoV-2 (COVID19) neutralizing antibody [Presence] in Serum by pVNT	LOINC
757679	SARS-CoV-2 (COVID19) neutralizing antibody [Titer] in Serum by pVNT	LOINC
757678	SARS-CoV-2 (COVID19) N gene [Presence] in Nose by NAA with probe detection	LOINC
757677	SARS-CoV-2 (COVID19) RNA [Presence] in Nose by NAA with probe detection	LOINC
756085	Measurement of Severe acute respiratory syndrome coronavirus 2 (SARS-CoV-2) using Nucleic acid amplification technique in Respiratory specimen	OMOP Extension
756084	Measurement of Severe acute respiratory syndrome coronavirus 2 (SARS-CoV-2) using Nucleic acid amplification technique in Unspecified specimen	OMOP Extension
756065	Measurement of Severe acute respiratory syndrome coronavirus 2 (SARS-CoV-2) in Unspecified specimen	OMOP Extension
756055	Measurement of Severe acute respiratory syndrome coronavirus 2 (SARS-CoV-2)	OMOP Extension
756029	Measurement of Severe acute respiratory syndrome coronavirus 2 (SARS-CoV-2) in Respiratory specimen	OMOP Extension

723480	SARS-CoV-2 (COVID19) Ab [Interpretation] in Serum or Plasma	LOINC
723479	SARS-CoV-2 (COVID19) IgG+IgM Ab [Presence] in Serum or Plasma by Immunoassay	LOINC
723478	SARS-CoV-2 (COVID19) ORF1ab region [Presence] in Respiratory specimen by NAA with probe detection	LOINC
723477	SARS-CoV-2 (COVID19) Ag [Presence] in Respiratory specimen by Rapid immunoassay	LOINC
723476	SARS-CoV-2 (COVID19) RNA [Presence] in Nasopharynx by NAA with non-probe detection	LOINC
723475	SARS-CoV-2 (COVID19) IgM Ab [Presence] in Serum or Plasma by Immunoassay	LOINC
723474	SARS-CoV-2 (COVID19) IgG Ab [Presence] in Serum or Plasma by Immunoassay	LOINC
723473	SARS-CoV-2 (COVID19) IgA Ab [Presence] in Serum or Plasma by Immunoassay	LOINC
723471	SARS-CoV-2 (COVID19) RdRp gene [Cycle Threshold #] in Respiratory specimen by NAA with probe detection	LOINC
723470	SARS-CoV-2 (COVID19) RdRp gene [Cycle Threshold #] in Unspecified specimen by NAA with probe detection	LOINC
723469	SARS-CoV-2 (COVID19) ORF1ab region [Cycle Threshold #] in Respiratory specimen by NAA with probe detection	LOINC
723468	SARS-CoV-2 (COVID19) S gene [Cycle Threshold #] in Unspecified specimen by NAA with probe detection	LOINC
723467	SARS-CoV-2 (COVID19) S gene [Cycle Threshold #] in Respiratory specimen by NAA with probe detection	LOINC
723466	SARS-CoV-2 (COVID19) S gene [Presence] in Unspecified specimen by NAA with probe detection	LOINC
723465	SARS-CoV-2 (COVID19) S gene [Presence] in Respiratory specimen by NAA with probe detection	LOINC
723464	SARS-CoV-2 (COVID19) ORF1ab region [Presence] in Unspecified specimen by NAA with probe detection	LOINC
723463	SARS-CoV-2 (COVID19) RNA [Presence] in Serum or Plasma by NAA with probe detection	LOINC
723459	SARS-CoV-2 (COVID19) IgA Ab [Units/volume] in Serum or Plasma by Immunoassay	LOINC
715272	SARS-CoV-2 (COVID19) N gene [Presence] in Nasopharynx by NAA with probe detection	LOINC
715262	SARS-CoV-2 (COVID19) RNA [Log #/volume] (viral load) in Unspecified specimen by NAA with probe detection	LOINC
715261	SARS-CoV-2 (COVID19) RNA [Presence] in Saliva (oral fluid) by Sequencing	LOINC
715260	SARS-CoV-2 (COVID19) RNA [Presence] in Saliva (oral fluid) by NAA with probe detection	LOINC
706181	SARS-CoV-2 (COVID19) IgG Ab [Presence] in Serum, Plasma or Blood by Rapid immunoassay	LOINC
706180	SARS-CoV-2 (COVID19) IgM Ab [Presence] in Serum, Plasma or Blood by Rapid immunoassay	LOINC
706179	SARS-CoV-2 (COVID19) Ab panel - Serum or Plasma by Immunoassay	LOINC

706 178	SARS-CoV-2 (COVID19) IgM Ab [Units/volume] in Serum or Plasma by Immunoassay	LOINC
706 177	SARS-CoV-2 (COVID19) IgG Ab [Units/volume] in Serum or Plasma by Immunoassay	LOINC
706 176	SARS-CoV-2 (COVID19) IgG and IgM panel - Serum, Plasma or Blood by Rapid immunoassay	LOINC
706 175	SARS-CoV-2 (COVID19) N gene [Presence] in Unspecified specimen by NAA with probe detection	LOINC
706 174	SARS-CoV-2 (COVID19) E gene [Presence] in Unspecified specimen by NAA with probe detection	LOINC
706 173	SARS-CoV-2 (COVID19) RdRp gene [Presence] in Unspecified specimen by NAA with probe detection	LOINC
706 170	SARS-CoV-2 (COVID19) RNA [Presence] in Unspecified specimen by NAA with probe detection	LOINC
706 169	SARS-CoV-2 (COVID19) RNA panel - Unspecified specimen by NAA with probe detection	LOINC
706 168	SARS-CoV-2 (COVID19) ORF1ab region [Cycle Threshold #] in Unspecified specimen by NAA with probe detection	LOINC
706 167	SARS-CoV-2 (COVID19) N gene [Cycle Threshold #] in Unspecified specimen by NAA with probe detection	LOINC
706 166	SARS-CoV-2 (COVID19) E gene [Cycle Threshold #] in Unspecified specimen by NAA with probe detection	LOINC
706 163	SARS-CoV-2 (COVID19) RNA [Presence] in Respiratory specimen by NAA with probe detection	LOINC
706 161	SARS-CoV-2 (COVID19) N gene [Presence] in Respiratory specimen by NAA with probe detection	LOINC
706 160	SARS-CoV-2 (COVID19) RdRp gene [Presence] in Respiratory specimen by NAA with probe detection	LOINC
706 158	SARS-CoV-2 (COVID19) RNA panel - Respiratory specimen by NAA with probe detection	LOINC
706 157	SARS-CoV-2 (COVID19) N gene [Cycle Threshold #] in Unspecified specimen by Nucleic acid amplification using CDC primer-probe set N1	LOINC
706 156	SARS-CoV-2 (COVID19) N gene [Presence] in Unspecified specimen by Nucleic acid amplification using CDC primer-probe set N1	LOINC
706 155	SARS-CoV-2 (COVID19) N gene [Cycle Threshold #] in Unspecified specimen by Nucleic acid amplification using CDC primer-probe set N2	LOINC
706 154	SARS-CoV-2 (COVID19) N gene [Presence] in Unspecified specimen by Nucleic acid amplification using CDC primer-probe set N2	LOINC
705 107	Measurement of Severe acute respiratory syndrome coronavirus 2 (SARS-CoV-2) in Sample from nose	OMOP Extension
705 106	Measurement of Severe acute respiratory syndrome coronavirus 2 (SARS-CoV-2) using Nucleic acid amplification technique in Sample from nose	OMOP Extension
705 001	Measurement of Severe acute respiratory syndrome coronavirus 2 (SARS-CoV-2) using Nucleic acid amplification technique	OMOP Extension
705 000	Measurement of Severe acute respiratory syndrome coronavirus 2 (SARS-CoV-2) using Nucleic acid amplification technique in Blood	OMOP Extension

704993	Measurement of Severe acute respiratory syndrome coronavirus 2 (SARS-CoV-2) using Sequencing	OMOP Extension
704992	Measurement of Severe acute respiratory syndrome coronavirus 2 (SARS-CoV-2) using Culture method	OMOP Extension
704991	Measurement of Severe acute respiratory syndrome coronavirus 2 (SARS-CoV-2) in Blood	OMOP Extension
700360	Infectious agent detection by nucleic acid (DNA or RNA); severe acute respiratory syndrome coronavirus 2 (SARS-CoV-2) (Coronavirus disease [COVID-19]), amplified probe technique	CPT4
586529	SARS-CoV-2 (COVID19) RNA [Cycle Threshold #] in Unspecified specimen by NAA with probe detection	LOINC
586528	SARS-CoV-2 (COVID19) RNA [Cycle Threshold #] in Respiratory specimen by NAA with probe detection	LOINC
586527	SARS-CoV-2 (COVID19) IgG Ab [Presence] in DBS by Immunoassay	LOINC
586526	SARS-CoV-2 (COVID19) RNA [Presence] in Nasopharynx by NAA with probe detection	LOINC
586525	SARS-CoV-2 (COVID19) N gene [Presence] in Respiratory specimen by Nucleic acid amplification using CDC primer-probe set N2	LOINC
586524	SARS-CoV-2 (COVID19) N gene [Presence] in Respiratory specimen by Nucleic acid amplification using CDC primer-probe set N1	LOINC
586523	SARS-CoV-2 (COVID19) E gene [Presence] in Respiratory specimen by NAA with probe detection	LOINC
586522	SARS-CoV-2 (COVID19) Ab [Units/volume] in Serum or Plasma by Immunoassay	LOINC
586521	SARS-CoV-2 (COVID19) IgA Ab [Presence] in Serum, Plasma or Blood by Rapid immunoassay	LOINC
586520	SARS-CoV-2 (COVID19) N gene [Presence] in Serum or Plasma by NAA with probe detection	LOINC
586519	SARS-CoV-2 (COVID19) S gene [Presence] in Serum or Plasma by NAA with probe detection	LOINC
586518	SARS-CoV-2 (COVID19) E gene [Presence] in Serum or Plasma by NAA with probe detection	LOINC
586517	SARS-CoV-2 (COVID19) whole genome [Nucleotide sequence] in Isolate by Sequencing	LOINC
586516	SARS-CoV-2 (COVID19) [Presence] in Unspecified specimen by Organism specific culture	LOINC
586515	SARS-CoV-2 (COVID19) Ab [Presence] in Serum or Plasma by Immunoassay	LOINC
586310	Measurement of Severe acute respiratory syndrome coronavirus 2 (SARS-CoV-2) Genetic material using Molecular method	OMOP Extension
586309	Measurement of Severe acute respiratory syndrome coronavirus 2 (SARS-CoV-2) in Specified specimen	OMOP Extension
586308	Measurement of Severe acute respiratory syndrome coronavirus 2 (SARS-CoV-2) using Nucleic acid amplification technique in Saliva	OMOP Extension

586307	Measurement of Severe acute respiratory syndrome coronavirus 2 (SARS-CoV-2) in Saliva	OMOP Extension

COVID-19 source codes

Id	Name	Vocabulary
45756093	Emergency use of U07.1 | COVID-19, virus identified	ICD10
45600471*	Other coronavirus as the cause of diseases classified elsewhere	ICD10CM
42501115	Emergency use of U07.1 | Disease caused by severe acute respiratory syndrome coronavirus 2	KCD7
710160	Emergency use of U07.1 | COVID-19, virus identified	ICD10CN
710159	COVID-19, excluding pneumonia	ICD10CN
710158	COVID-19	ICD10CN
710156	COVID-19 pneumonia	ICD10CN
710155	COVID-19 pneumonia	ICD10CN
586415*	Provisional assignment of new diseases or emergency use	KCD7
586414*	Novel coronavirus infection	KCD7

*Omitted for CUIMC to avoid including non-COVID-19 patients.
