# Supplementary material for: Deep phenotyping of 34,128 adult patients hospitalised with COVID-19 in an international network study
Source: Nat Commun. 2020 Oct 6;11:5009. doi: 10.1038/s41467-020-18849-z (PMC7538555; doi:10.1038/s41467-020-18849-z)
Supplement: Supplementary file 3 — Reporting Summary [file 41467_2020_18849_MOESM3_ESM.pdf]

nature research

Corresponding author(s): Daniel Prieto-Alhambra

Last updated by author(s): Aug 31, 2020

Reporting Summary

Nature Research wishes to improve the reproducibility of the work that we publish. This form provides structure for consistency and transparency in reporting. For further information on Nature Research policies, see our Editorial Policies and the Editorial Policy Checklist.

Statistics

For all statistical analyses, confirm that the following items are present in the figure legend, table legend, main text, or Methods section.

n/a	Confirmed	
☐	☒	The exact sample size (*n*) for each experimental group/condition, given as a discrete number and unit of measurement
☒	☐	A statement on whether measurements were taken from distinct samples or whether the same sample was measured repeatedly
☒	☐	The statistical test(s) used AND whether they are one- or two-sided<br>*Only common tests should be described solely by name; describe more complex techniques in the Methods section.*
☐	☒	A description of all covariates tested
☒	☐	A description of any assumptions or corrections, such as tests of normality and adjustment for multiple comparisons
☒	☐	A full description of the statistical parameters including central tendency (e.g. means) or other basic estimates (e.g. regression coefficient) AND variation (e.g. standard deviation) or associated estimates of uncertainty (e.g. confidence intervals)
☒	☐	For null hypothesis testing, the test statistic (e.g. *F*, *t*, *r*) with confidence intervals, effect sizes, degrees of freedom and *P* value noted<br>*Give P values as exact values whenever suitable.*
☒	☐	For Bayesian analysis, information on the choice of priors and Markov chain Monte Carlo settings
☒	☐	For hierarchical and complex designs, identification of the appropriate level for tests and full reporting of outcomes
☐	☒	Estimates of effect sizes (e.g. Cohen's *d*, Pearson's *r*), indicating how they were calculated

Our web collection on statistics for biologists contains articles on many of the points above.

Software and code

Policy information about availability of computer code

Data collection	All sites that contributed to the study had mapped their source data to the OMOP common data model. A bespoke study package was developed to extract aggregated patient characteristics from each of the sites, without the need for the sharing of patient-level data itself. The code used is available here: https://github.com/ohdsi-studies/Covid19HospitalizationCharacterization
Data analysis	The data analysis was performed in R 4.0.0, using a bespoke study package which is available here https://github.com/ohdsi-studies/Covid19HospitalizationCharacterization

For manuscripts utilizing custom algorithms or software that are central to the research but not yet described in published literature, software must be made available to editors and reviewers. We strongly encourage code deposition in a community repository (e.g. GitHub). See the Nature Research guidelines for submitting code & software for further information.

Data

Policy information about availability of data

All manuscripts must include a data availability statement. This statement should provide the following information, where applicable:

- Accession codes, unique identifiers, or web links for publicly available datasets
- A list of figures that have associated raw data
- A description of any restrictions on data availability

The analytic code used in the study has been made available at https://github.com/ohdsi-studies/Covid19HospitalizationCharacterization. This study package is run from R and is a OHDSI CohortDiagnostics-type package.[15] This makes use of other OHDSI packages, such as FeatureExtraction,[16] to extract patient characteristics for user-specified cohorts in the common data model. The aggregated results set, that does not include patient-level health information, is available via http://evidence.ohdsi.org/Covid19CharacterizationHospitalization/. Data partners (Columbia University Irving Medical Center [CUIMC], Health Insurance Review & Assessment [HIRA], HM Hospitales [HM], Premier Hospital database [PHD], The Information System for Research in Primary Care [SIDIAP], United States

Department of Veterans Affairs [VA OMOP], and University of Colorado Health Data Compass [UC HDC]) contributing to this study remain custodians of their individual patient-level health information.

Field-specific reporting

Please select the one below that is the best fit for your research. If you are not sure, read the appropriate sections before making your selection.

- [x] Life sciences
- [ ] Behavioural & social sciences
- [ ] Ecological, evolutionary & environmental sciences

For a reference copy of the document with all sections, see nature.com/documents/nr-reporting-summary-flat.pdf

Life sciences study design

All studies must disclose on these points even when the disclosure is negative.

Sample size	All individuals who were eligible to be included across the participating sites were included in the study.
Data exclusions	As pre-specified in the protocol, where less than 10 individuals contributed a particular characteristic then this characteristic has not been reported so as to maintain patient confidentiality.
Replication	N/A (this was an observational study describing the characteristics of patients hospitalised with COVID-19)
Randomization	N/A (this was an observational study describing the characteristics of patients hospitalised with COVID-19)
Blinding	N/A (this was an observational study describing the characteristics of patients hospitalised with COVID-19)

Reporting for specific materials, systems and methods

We require information from authors about some types of materials, experimental systems and methods used in many studies. Here, indicate whether each material, system or method listed is relevant to your study. If you are not sure if a list item applies to your research, read the appropriate section before selecting a response.

Materials & experimental systems

n/a	Involved in the study	
x		Antibodies
x		Eukaryotic cell lines
x		Palaeontology and archaeology
x		Animals and other organisms
	x	Human research participants
x		Clinical data
x		Dual use research of concern

Methods

n/a	Involved in the study	
x		ChIP-seq
x		Flow cytometry
x		MRI-based neuroimaging

Human research participants

Policy information about studies involving human research participants

Population characteristics	Patients hospitalised with COVID-19 aged 18 or over
Recruitment	Patients hospitalised between December 2019 and April 2020 with COVID-19 were identified on the basis of having a hospitalisation along with a confirmatory diagnosis or test result of COVID-19 within a time window from 21 days prior to admission up to the end of their hospitalisation. Data from the US, South Korea, and Spain underpinned the study. EHR data from the US came from the Columbia University Irving Medical Center (CUIMC), covering NewYork-Presbyterian Hospital/ Columbia University Irving Medical Center, University of Colorado Health Data Compass (UC HDC), which includes the UCHealth System with data from 12 hospitals, and United States Department of Veterans Affairs (VA OMOP), which includes 170 medical centers. In addition, data from a US hospital billing system database came from the Premier Hospital database (PHD). EHR data from Spain came from The Information System for Research in Primary Care (SIDIAP), a primary care records database that covers approximately 80% of the population of Catalonia, Spain, and the inpatient care database of HM Hospitales (HM), a hospital group which includes fifteen general hospitals from all over Spain, with detailed hospital admission information for COVID-19 patients from March 1st to April 20th 2020. Data from South Korea came from Health Insurance Review & Assessment (HIRA), a repository of national claims data which is collected in the process of reimbursing healthcare providers.
Ethics oversight	All the data partners received Institutional Review Board (IRB) approval or exemption. STARR-OMOP had approval from IRB Panel #8 (RB-53248) registered to Leland Stanford Junior University under the Stanford Human Research Protection Program (HRPP). The use of VA data was reviewed by the Department of Veterans Affairs Central Institutional Review Board (IRB) and was determined to meet the criteria for exemption under Exemption Category 4(3) and approved the request for Waiver of HIPAA Authorization. The research was approved by the Columbia University Institutional Review Board as an OHDSI network study. The IRB number for use of HIRA data was AJIB-MED-EXP-20-065. HM Hospitales and SIDIAP analyses were approved by the Clinical Research Ethics Committee of the IDIAPJGol (project code: 20/070-PCV). The UC-HDC data use was reviewed by Colorado Multi-Institutional Review Board (COMIRB) and was determined to meet the criteria for exemption under Exemption Category 4(3) and approved the request for Waiver of HIPAA Authorization (protocol # 20-0730).

Note that full information on the approval of the study protocol must also be provided in the manuscript.
